# Dedifferentiated Liposarcoma Arising in an Esophageal Polyp: A Case Report

**DOI:** 10.1155/2012/141693

**Published:** 2012-08-14

**Authors:** Jorge Torres-Mora, Ann Moyer, Mark Topazian, Jeffrey Alexander, Tsung-Teh Wu, Amber Seys, Karen Fritchie

**Affiliations:** ^1^Department of Laboratory Medicine and Pathology, Mayo Clinic, Rochester, MN 55905, USA; ^2^Department of Gastroenterology, Mayo Clinic, Rochester, MN 55905, USA

## Abstract

Liposarcoma is one of the most common sarcomas in adults, but only rarely presents as an esophageal primary. There have been several reports of well-differentiated liposarcoma (WDL) arising in the esophagus, but we present a case of dedifferentiated liposarcoma (DL) presenting as a large esophageal polyp. We believe this is the first reported case of DL of the esophagus with morphologic evidence of both well-differentiated and dedifferentiated components. The diagnosis was confirmed by demonstration of CPM gene amplification by fluorescence in situ hybridization (FISH).

## 1. Introduction

Liposarcoma is one of the most common sarcomas in adults [1]. Liposarcomas can be divided into four main subtypes: WDL/DL, myxoid/round cell liposarcoma, and pleomorphic liposarcoma. However, since WDL and DL share clinical, histologic, and cytogenetic features, it is easiest to think of these entities as different ends of a morphologic spectrum. While it is not uncommon to encounter WDL and DL in the retroperitoneum, these tumors are exceedingly uncommon in the esophagus. Here we report the interesting case of a DL presenting as a large esophageal polyp.

## 2. Report

An 81-year-old male presented to clinic with a 2-year history of an esophageal polyp first detected on screening endoscopy. Biopsies performed at that time were reported to be benign, and the decision was made to follow the lesion. He was relatively asymptomatic until he began experiencing trouble swallowing after a recent upper respiratory tract infection two weeks before. Physical examination revealed a well-developed, well-nourished male without any obvious oral cavity masses or abnormalities detected during routine evaluation. Flexible laryngoscopy was performed to evaluate the nasopharynx, oropharynx, hypopharynx, and larynx but showed only an elongated uvula. Computed tomography (CT) showed a large intraluminal mass extending into the upper esophagus. The imaging features were consistent with a fatty mass with an enhancing component, most suggestive of a fibrovascular polyp. The patient had no other significant past medical history and no prior history of malignancy.

Because the patient was experiencing symptoms secondary to the mass, the decision was made to resect the mass endoscopically. Intraoperative findings included a lengthy polypoid lesion covered by unremarkable mucosa arising near the distal margin of the upper esophageal sphincter and extending 16 to 28 cm from the incisors. The duodenum, stomach, and remaining esophagus appeared normal. Endoscopic ultrasound confirmed that the mass did not involve the deep wall layers of the esophagus. The mass was resected at its base.

**Figure 1:**
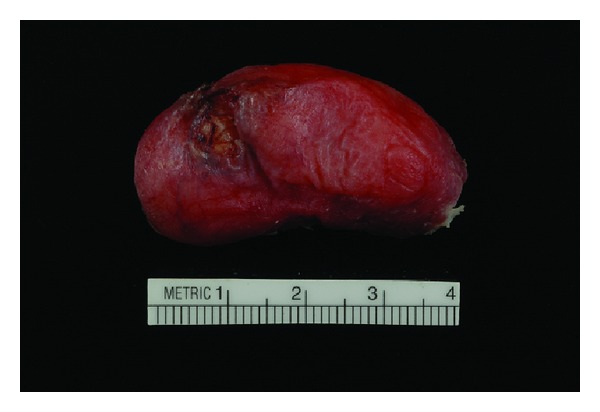
Gross examination reveal pink-tan tissue fragments.

**Figure 2:**
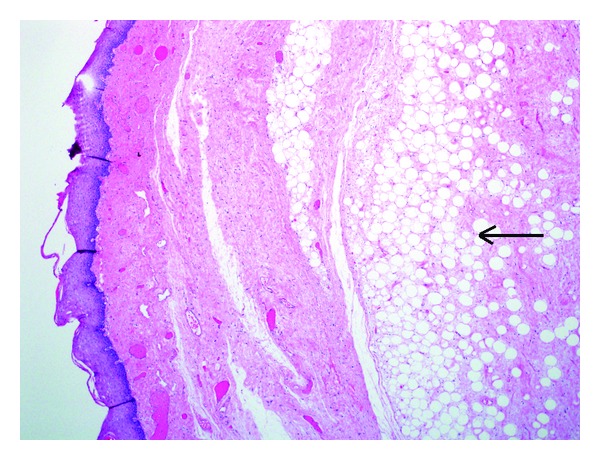
Area of well-differentiated fatty component (arrow). The lesion is submucosal.

**Figure 3:**
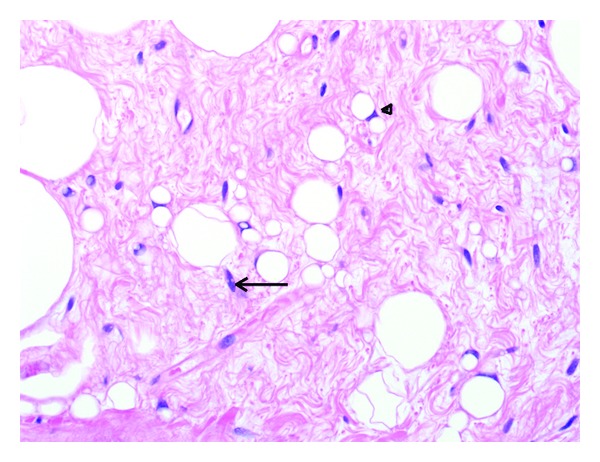
Examination of the fibrous septa shows atypical hyperchromatic stromal cells (arrow) and scattered lipoblasts (arrowhead).

**Figure 4:**
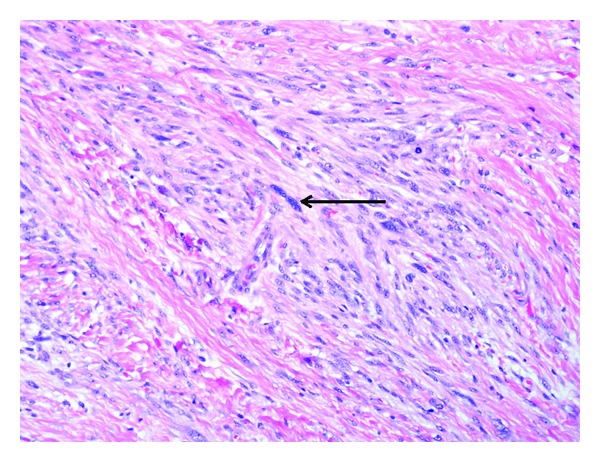
The high-grade component was composed of atypical spindle cells with nuclear hyperchromasia.

**Figure 5:**
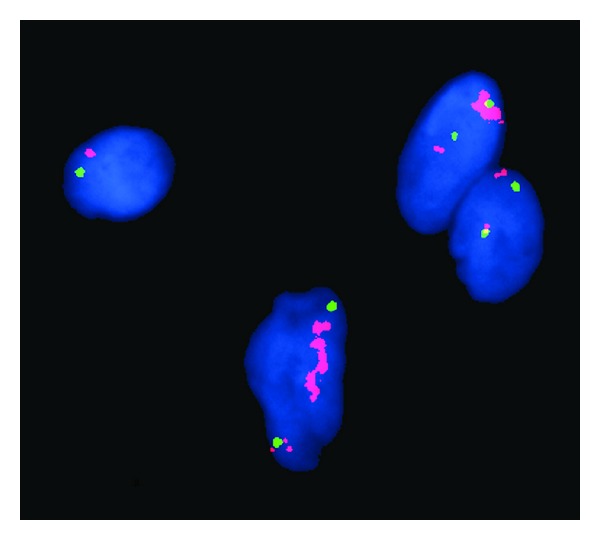
Fluorescence in situ hybridization studies showed amplification of the *CPM* gene.

Gross examination revealed two portions of pink-tan tissue measuring 7.3 × 2.8 × 1.4 cm and 4.5 × 2.8 × 1.2 cm (Figure 1). Sectioning of the larger fragment showed a 2.7 cm firm region. Microscopic examination revealed a biphasic submucosal neoplasm (Figure 2) composed of a well-differentiated lipomatous component adjacent to a high-grade spindle cell sarcoma. Examination of the well-differentiated areas showed mature adipose tissue with fibrous septae containing atypical hyperchromatic stromal cells and scattered lipoblasts (Figure 3) which involved the surgical resection margin. The morphologically high-grade component corresponded to the firm area identified grossly and was composed of atypical spindle cells with nuclear hyperchromasia and easily identifiable mitotic figures (Figure 4). The spindle cells were negative for cytokeratin OSCAR, smooth muscle actin, desmin, KIT, and S100. Fluorescence in situ hybridization studies showed amplification of the *CPM* gene (Figure 5). The morphologic and molecular findings supported the diagnosis of DL.

One month after the resection of the tumor, the patient reports that his swallowing is almost back to normal. Upper endoscopy performed at this time showed mild edema of the right pyriform fossa mucosa and a 1 cm hypoechoic right paratracheal lymph node. Fine-needle aspiration of this lymph node was negative for malignancy. There was no evidence of recurrent or residual tumor. The patient was offered radiation therapy or close endoscopic followup and is undecided at this time.

## 3. Discussion

DLs are biphasic malignant adipocytic tumors that contain areas of WDL with transition to nonlipogenic sarcoma [2]. These tumors typically develop in older adults (sixth to seventh decades) with an equal distribution between males and females [1]. The most common location of this tumor is the retroperitoneum, although less common sites include groin, deep soft tissue of the extremity and mediastinum. DL can occur de novo (90%) or may arise from a preexisting WDL (10%) [3]. The genetic hallmark of these tumors is giant marker or supernumerary ring chromosomes that contain amplification of chromosome 12q13–15, a finding also characteristic of WDL [1, 4]. This region of chromosome 12 contains genes such as *MDM2*, *CDK4*, and *HMGIC.* More recently, the *FRS2* gene in 12q15 has also been shown to be amplified in this setting [5]. The *CPM* gene (carboxypeptidase M) is telomeric to the *MDM2* locus on chromosome 12q15 and is consistently coamplified with *MDM2*, allowing it's use as a surrogate to *MDM2 *[6].

Mansour et al. described the first report of a primary liposarcoma of the esophagus in 1983 [7]. Since that time approximately twenty additional cases have been reported in the English literature. In 2011 Watkin et al. reported the first case of DL of the esophagus with molecular confirmation [8]. However, the case described by Watkin consisted only of a high-grade pleomorphic and spindle cell proliferation without morphologic evidence of a WDL component. Although the study of such tumor by CGH showed a simple genomic profile with only a few amplifications which is suggestive of DL [9], the presence of *MDM2* gene amplification *per se* cannot be equated with the presence of a DL, since MDM2 gene amplification by FISH can be identified in up to 40% of pleomorphic sarcomas [10]. Therefore, it remains uncertain if the tumor that Watkin described was a DL or a pleomorphic sarcoma with *MDM2* amplification.

The behavior of DL is slightly better than other high-grade pleomorphic sarcomas [1]. However, local recurrences are common, and these tumors may metastasize. Surgical excision is the treatment of choice, while the roles of chemotherapy and radiation remain controversial. Regardless of the use of adjuvant therapy, careful long-term followup is warranted.

Liposarcoma of the esophagus is rare but should be considered in the differential diagnosis of large esophageal masses or polyps. We believe that our case represents the first report of DL of the esophagus with histologic documentation of both well-differentiated and dedifferentiated components, as well as confirmation with molecular analysis.
